# TXNL1 in redox and protein quality control: mechanisms and disease relevance

**DOI:** 10.3389/fcell.2026.1876585

**Published:** 2026-08-18

**Authors:** Shanshan Wu, Deyuan Li, Dongqiong Xiao

**Affiliations:** 1 Department of Emergency/Key Laboratory of Birth Defects and Related Diseases of Women and Children (Ministry of Education), West China Second University Hospital, Sichuan University, Chengdu, China; 2 Department of Pediatrics/Key Laboratory of Birth Defects and Related Diseases of Women and Children (Ministry of Education), West China Second University Hospital, Sichuan University, Chengdu, China; 3 Chengdu Hi-Tech Zong Hospital for Women and Children, Chengdu, China

**Keywords:** chaperone function, oxidative stress, protein degradation, redox, TXNL1 (thioredoxin-like protein 1)

## Abstract

TXNL1 (thioredoxin-like protein 1), a member of the thioredoxin superfamily, integrates thioredoxin oxidoreductase activity with molecular chaperone function. Through its N-terminal TRX domain and C-terminal PITH domain, TXNL1 not only helps maintain cellular redox homeostasis but also prevents toxic aggregation of oxidatively damaged proteins. Consequently, TXNL1 acts as a critical buffer in the cellular defense against oxidative stress and in protein quality control. TXNL1 is widely expressed across diverse tissues, and its dysregulation is closely associated with multiple pathological conditions, including chemotherapy resistance in gastric cancer, poor prognosis in colorectal cancer, arsenic-induced malignant transformation of bronchial epithelial cells, and ischemic as well as traumatic injuries of the central nervous system. TXNL1 undergoes degradation through both canonical ubiquitin-dependent and ubiquitin-independent mechanisms. This review summarizes the discovery, molecular architecture, expression patterns, physiological functions, and disease-related research progress of TXNL1, and discusses its potential clinical applications as a biomarker and therapeutic target.

## 1 Introduction

The cellular antioxidant defense system is a complex network responsible for scavenging reactive oxygen species (ROS) and reactive nitrogen species (RNS) to maintain redox homeostasis. This network comprises three major components: enzymatic antioxidants, non-enzymatic antioxidants (such as glutathione and vitamins C/E), and exogenous antioxidants (e.g., dietary polyphenols). The enzymatic antioxidant system, which includes catalase, superoxide dismutase, glutathione peroxidase, thioredoxin, and peroxiredoxin, plays a pivotal role in this process (Alhaj Sulaiman and Katanaev, 2025).

The thioredoxin system, first identified in *Escherichia coli* in 1964, consists of thioredoxin (Trx) and thioredoxin reductase (TrxR) and is central to controlling cellular redox balance (Alhaj Sulaiman and Katanaev, 2025). Members of the thioredoxin (Trx) superfamily share a conserved structural fold characterized by a four-stranded β-sheet flanked by three α-helices, featuring a hallmark dithiol Cys-x-x-Cys (CxxC) active site at the N-terminus of the first α-helix. These proteins perform essential functions in antioxidant defense, protein quality control, ribonucleotide reductase activation, and transcription factor regulation (Atkinson and Babbitt, 2009).

Thioredoxin-like protein 1 (TXNL1) is a member of the thioredoxin (Trx) family characterized by a conserved dithiol CxxC active site motif (CGPC), which is consistent with the catalytic mechanism of classical thioredoxin. The *TXNL1* gene was initially identified in 1997 from a 421 bp EST (AA745861) from a human brain library, encoding a Trx-like peptide. Within 1 year, two research groups independently cloned the full-length 1 kb cDNA, designated *TRP32* (AJ000542 and AB005582) (Lee et al., 1998; Miranda-Vizuete et al., 1998). This gene, now officially designated *TXNL1* by the HUGO Gene Nomenclature Committee, is located on chromosome 18q21.31. TXNL1 is highly conserved across species and exhibits a broad expression range from lower unicellular eukaryotes, such as yeast, to mammals (Andor et al., 2023).

Accumulating evidence indicates that TXNL1 plays key roles in protein quality control and the indirect regulation of oxidative stress (Andor et al., 2023). Although TXNL1 cannot directly scavenge ROS (Zhao et al., 2025), it counteracts oxidative stress through multiple mechanisms. First, TXNL1 repairs oxidatively damaged proteins via its TRX domain, a function that has been experimentally demonstrated (Andor et al., 2023). Second, TXNL1 exhibits chaperone activity, preventing the aggregation and precipitation of reduced protein substrates (Andor et al., 2023). Third, TXNL1 interacts with the proteasome via its PITH domain (Andersen et al., 2009; Arkinson et al., 2025; Gao et al., 2025); however, direct evidence supporting a role of this interaction in the degradation of oxidatively damaged proteins remains to be established. Beyond these functions, TXNL1 may act as a redox sensor that couples intracellular redox signals to specific membrane transport events (Felberbaum-Corti et al., 2007).

Notably, altered expression levels of TXNL1 have been observed in various diseases (Zhao and Qi, 2021), particularly in the context of tumor drug resistance and neurological injury disorders. Consequently, TXNL1 is emerging as a potential therapeutic target for these conditions.

## 2 Structure of TXNL1

Molecular cloning identified the full-length *TXNL1* cDNA of 1,278 bp, encoding 289 amino acid residues (Miranda-Vizuete et al., 1998). The protein consists of two distinct domains: an N-terminal thioredoxin domain (TRX domain, residues 2-109) and a C-terminal PITH domain (residues 115–285; originally termed DUF1000, PFAM: pfam06201), connected by a flexible linker and followed by a short C-terminal tail (Arkinson et al., 2025).

The N-terminal Trx domain adopts a classical thioredoxin fold, which endows TXNL1 with reductase activity (Andor et al., 2023). This reversible redox mechanism is characteristic of thioredoxin family proteins and is essential for TXNL1’s ability to sense and respond to changes in the cellular redox state.

The C-terminal PITH domain is predominantly composed of β-sheet structures (Andor et al., 2023). As a module that interacts with the proteasome, it enables TXNL1 to participate in the proteasome regulatory network as a cofactor (Arkinson et al., 2025; Gao et al., 2025).

## 3 Expression and distribution of TXNL1

TXNL1 exhibits widespread and readily detectable expression in human tissues and organs (Miranda-Vizuete et al., 1998). Data from the Human Protein Atlas classify *TXNL1* as a housekeeping gene, albeit with tissue-specific variation in expression levels. The highest expression occurs in the thyroid, moderate expression in the testis (male) and gallbladder, and relatively low expression in the cerebral cortex (Miranda-Vizuete et al., 1998). In contrast to the human expression profile, Andersen et al. reported comparable TXNL1 protein levels across various mouse tissues—including the heart, spleen, liver, lung, brain, and kidney, as determined by Western blot analysis (Andersen et al., 2009).

The mRNA expression profile of *TXNL1* varies considerably among non-mammalian species (Liyanage et al., 2018; Mo et al., 2023). In the Chinese giant salamander (*Andrias davidianus*), *TXNL1* mRNA is most abundantly expressed in the liver, with widespread distribution across other tissues (Mo et al., 2023). In contrast, in the big-belly seahorse (*Hippocampus abdominalis*), *TXNL1* mRNA is most highly expressed in muscle tissue, followed by the ovary, brain, gills, and blood (Liyanage et al., 2018). Immune stimulation can induce time-dependent changes in *TXNL1* expression: following challenge with *Aeromonas hydrophila*, the transcriptional level of *TXNL1* in the liver of the Chinese giant salamander was significantly upregulated (Mo et al., 2023); similarly, stimulation with *Edwardsiella tarda* and *Streptococcus iniae* induced significant time-dependent expression of *TXNL1* mRNA in the gills and blood of the big-belly seahorse (Liyanage et al., 2018).

Existing studies have demonstrated that subcellular localization studies indicate that TXNL1 is a soluble protein distributed in both the cytoplasm and nucleus (Andersen et al., 2009; Dilshan et al., 2023). GFP fusion protein fluorescence microscopy demonstrated this distribution and subcellular fractionation in HeLa cells. TXNL1 is not secreted into the extracellular space and remains undetectable in the cell culture medium (Andersen et al., 2009). Future research should utilize advanced imaging approaches, such as immunoelectron microscopy and live-cell imaging, to better characterize the dynamic shuttling of TXNL1 between the cytoplasm and nucleus. Moreover, the dynamic behavior of TXNL1 under various oxidative stress conditions requires further detailed exploration.

## 4 Function of TXNL1

### 4.1 Protein quality control

#### 4.1.1 Thioredoxin-like redox activity and facilitation of protein degradation

TXNL1 exhibits typical thioredoxin-like oxidoreductase activity dependent on its conserved CGPC active site motif (redox potential of approximately-250 mV), catalyzing the reduction of disulfide bonds in substrates such as insulin, cystine, and oxidized glutathione (GSSG) (Andor et al., 2023). This reductase activity is similar to that of thioredoxin (TXN/Trx1) and is dependent on the CxxC redox-active motif. Andersen et al. utilized an active-site cysteine-to-serine mutant to capture transient disulfide intermediates during the reduction process, identifying eEF1A1 (eukaryotic translation elongation factor 1A1) as a potential physiological substrate of TXNL1 (Andersen et al., 2009).

**FIGURE 1:**
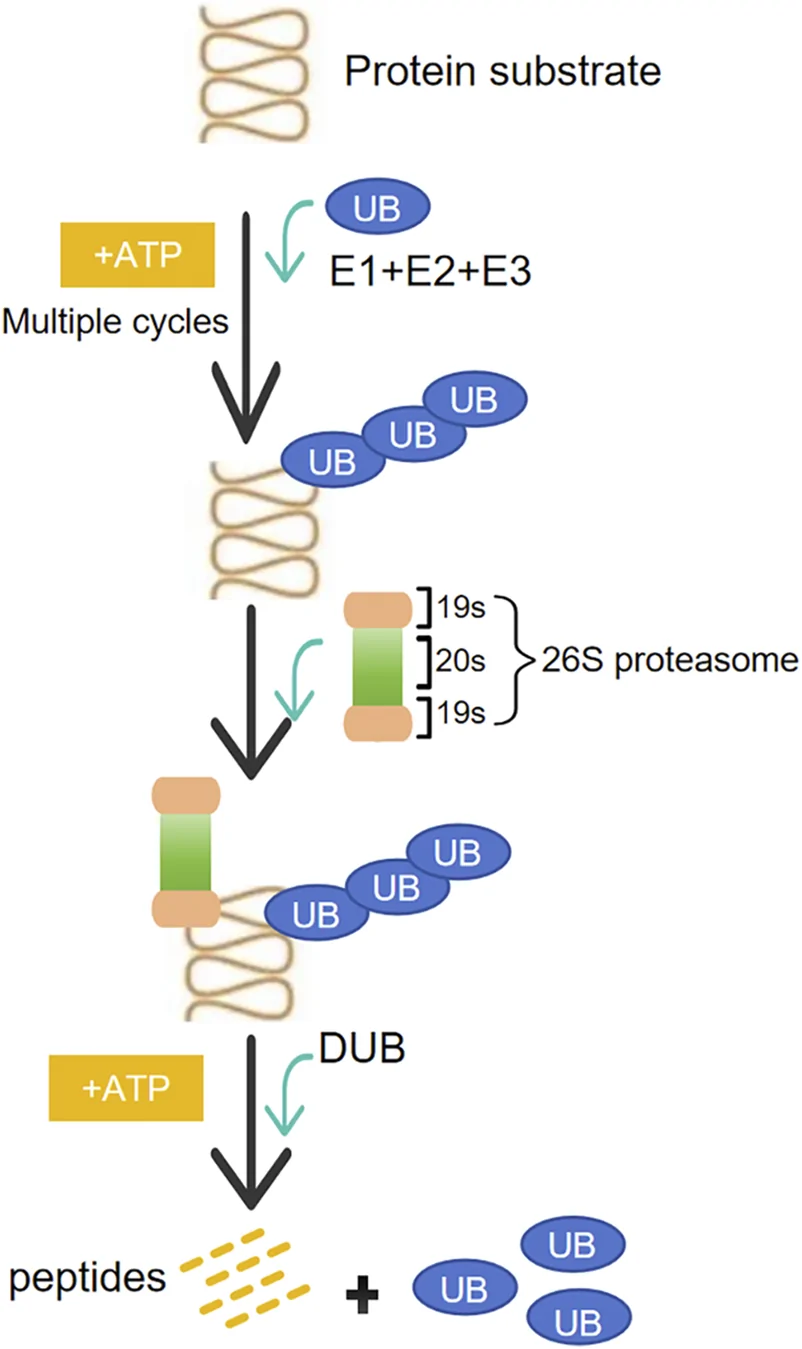
The classical ubiquitination pathway. Ubiquitin (Ub) is activated in an ATP-dependent reaction and transferred via a sequential cascade comprising the E1 ubiquitin-activating enzyme, E2 ubiquitin-conjugating enzyme, and E3 ubiquitin ligase. This process results in the covalent conjugation of Ub to a substrate protein, forming a polyubiquitin chain. This polyubiquitin tag is specifically recognized by the 19S regulatory particle of the 26S proteasome, leading to substrate unfolding and subsequent proteolytic degradation into short peptides by the 20S core particle. Concurrently, deubiquitinases (DUBs) cleave ubiquitin moieties for recycling in subsequent catalytic cycles.

The ubiquitin-proteasome pathway represents the central selective protein degradation system in eukaryotic cells (Liao et al., 2024) (Figure 1). This ATP-dependent mechanism involves the covalent attachment of ubiquitin molecules to substrate proteins via a cascade of E1 activating enzymes, E2 conjugating enzymes, and E3 ligases. Polyubiquitin chain formation subsequently mediates substrate recognition and degradation by the 26S proteasome (Hershko and Ciechanover, 1998). The 26S proteasome is a large protease complex comprising a proteolytically active 20S core particle and one or two 19S regulatory particles. The 19S regulatory particle recognizes ubiquitinated substrates, mediates deubiquitination and substrate unfolding, and translocates substrates into the 20S core particle for degradation. The 19S regulatory particle is assembled from about 19 distinct subunits, with PSMD1 (Rpn2), PSMD4 (Rpn10), and PSMD14 (Rpn11) serving as structurally and functionally essential components (Arkinson et al., 2025).

**FIGURE 2:**
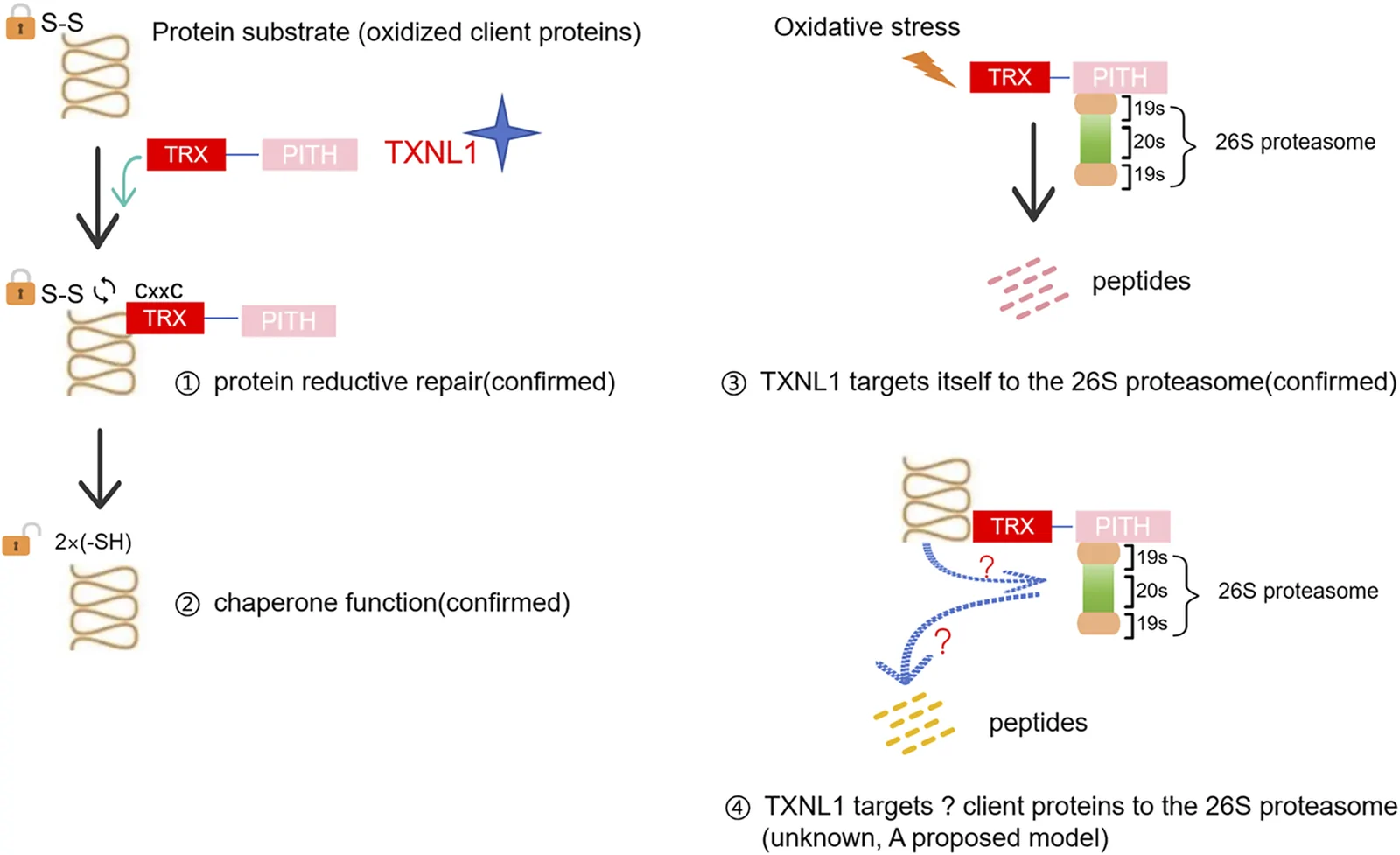
TXNL1 in redox and protein quality control: Ubiquitin-independent proteasomal degradation pathway. TXNL1 functions as a redox-active cofactor, integrating thioredoxin oxidoreductase activity with proteasome regulatory functions. TXNL1 utilizes its thioredoxin (TRX) domain, containing a CxxC motif, and its PITH domain to mediate reductive repair (①) or chaperone functions (②) on oxidized client proteins. Upon exposure to oxidative stress (e.g., arsenite), TXNL1 undergoes conformational changes and binds directly to the 19S regulatory particle of the 26S proteasome. This interaction facilitates the ubiquitin-independent degradation of TXNL1 itself (③), a mechanism substantiated by structural and functional evidence. However, whether TXNL1 concurrently targets bound client proteins for co-degradation (④) remains to be elucidated.

In contrast to the classical ubiquitin-dependent degradation system—a non-specific, ATP-dependent, ubiquitin-driven bulk clearance machinery that maintains basal proteostasis in eukaryotic cells—the degradation of TXNL1 proceeds via a distinct ubiquitin-independent pathway (Figure 2). It is currently thought that this pathway is triggered by metal/metalloid oxidant-induced oxidative stress (e.g., arsenic exposure). This mechanism expands our understanding of substrate recognition by the proteasome. Andersen et al. demonstrated that TXNL1 enhances the efficiency of proteasomal degradation, as knockdown of *TXNL1* in HEK293 cells resulted in decreased degradation of ubiquitinated protein substrates (Andersen et al., 2009). Under oxidative stress conditions (e.g., arsenite treatment), TXNL1 undergoes conformational changes and binds directly to the 19S regulatory particle of the 26S proteasome. This interaction leads to the ubiquitin-independent degradation of TXNL1 itself (Andersen et al., 2009; Arkinson et al., 2025; Gao et al., 2025). Whether this pathway also functions under oxidative stress conditions other than those elicited by metal/metalloid oxidants remains to be determined. Additionally, Whether TXNL1 also co-targets its bound client proteins for degradation remains unclear and requires further investigation.

TXNL1 binds to the 19S regulatory particle and functions as a cofactor of the 26S proteasome (Andersen et al., 2009; Arkinson et al., 2025; Gao et al., 2025). In 2025, Gao et al. (2025) used cryo-electron microscopy to resolve the structure of the TXNL1-19S regulatory particle complex, revealing direct interaction interfaces between TXNL1 and three subunits of the 19S regulatory particle (PSMD1, PSMD4, and PSMD14). Furthermore, this study revealed that the binding constitutes the structural prerequisite for ubiquitin-independent degradation of TXNL1 under oxidative stress conditions. Another study (Arkinson et al., 2025) published concurrently resolved distinct binding modes of TXNL1 with the 26S proteasome, revealing that TXNL1 does not associate with the proteasome statically but rather acts as a “molecular switch” that adjusts its binding position (particularly the occlusion of the Rpn11 active site by its C-terminus) and its affinity for the proteasome depending on whether the proteasome is in a resting or active (degrading) state. In the resting-state proteasome, TXNL1 binds with low affinity at an alternative position above the Rpn11 deubiquitinase. In contrast, upon substrate (mEos3.2) binding-triggered conformational activation of the proteasome, the C-terminal tail of TXNL1 occludes the catalytic groove of Rpn11 and coordinates the active-site Zn^2+^ ion. This, together with TXNL1’s binding to PSMD1 (Rpn2), PSMD4 (Rpn10), and PSMD14 (Rpn11) within the 19S regulatory particle, collectively constitutes a high-affinity conformational binding to the proteasome, allowing TXNL1 to function as a conformation-dependent proteasome cofactor in the proteasome regulatory network.

#### 4.1.2 Chaperone function

Molecular chaperones are a class of highly conserved proteins that recognize and bind to hydrophobic regions of non-native conformational substrates (such as partially folded, misfolded, or denatured proteins), thereby inhibiting their abnormal aggregation, maintaining protein homeostasis, and facilitating proper folding or targeted degradation. They are essential for cellular stress responses and protein quality control (Fernández-Fernández et al., 2016; Gu et al., 2025). ATP-independent molecular chaperones can rapidly capture denatured proteins without energy input, serving as the “first line of defense” in cellular stress responses. Small heat shock proteins (sHSPs) are a typical example, capable of forming dynamic complexes with various non-native conformational proteins in an ATP-independent manner to prevent their irreversible aggregation (Mogk and Bukau, 2017).

The ATP-independent molecular chaperone function of TXNL1 has been well established: TXNL1 can effectively prevent the aggregation and precipitation of substrates in non-native conformations resulting from disulfide bond disruption (such as reduced insulin generated by reducing agent treatment) under heating conditions, and can also protect proteins in whole-cell lysates from heat-induced aggregation, all without requiring ATP. The chaperone activity of TXNL1 is entirely independent of its redox function, as Cys-to-Ser mutants retain activity in the absence of TrxR1 and NADPH, confirming that its chaperone function does not rely on catalytic cysteine residues or disulfide exchange reactions, and is unrelated to the redox catalytic pathway (Andor et al., 2023).

However, the specific domain, key amino acid residues, oligomeric state, or dynamic conformational features through which TXNL1 exerts its chaperone function have not yet been clearly elucidated, and it is only speculated that TXNL1 may bind to its substrate (reduced insulin) via non-covalent interactions (Andor et al., 2023). Reduced insulin, due to disulfide bond cleavage, undergoes partial structural unfolding, exposing previously buried hydrophobic regions, which readily leads to irreversible aggregation and precipitation through hydrophobic interactions (Dolui et al., 2025). It is speculated that TXNL1 may form dynamic, reversible non-covalent complexes with these exposed regions through surface complementary areas (such as hydrophobic surfaces and dynamic loop regions), thereby ‘shielding' insulin in solution and preventing precipitation.

Insulin is subjected to diverse and multi-layered chaperone-mediated regulation, involving chaperones in both the endoplasmic reticulum and the cytoplasm (Podraza-Farhanieh et al., 2020; Viviano et al., 2020). Current evidence for TXNL1 is primarily derived from the *in vitro* reduced insulin model (Andor et al., 2023), and its direct role in the physiological folding pathway of insulin remains unclear. However, based on its chaperone function, we speculate that TXNL1 may participate in the maintenance of insulin homeostasis under stress conditions. In addition to reduced insulin, TXNL1 also inhibits the aggregation of multiple proteins in whole-cell lysates under heat stress (Andor et al., 2023). This protective effect on whole-cell lysate proteins suggests that TXNL1 may function as a general 'holdase'-type chaperone for a broad range of proteins, although its full spectrum of target proteins remains incompletely defined. In summary, the *in vitro* chaperone function of TXNL1 as an ATP-independent molecular chaperone has been validated, yet its structural basis, physiological networks, and disease-related mechanisms urgently warrant in-depth investigation.

### 4.2 Participation in the response to oxidative stress

Under oxidative stress, various triggers-including metal/metalloid oxidants, hydrogen peroxide (H_2_O_2_), and ischemia-can perturb cellular redox homeostasis, leading to excessive reactive oxygen species (ROS) production. These ROS further mediate inflammatory, apoptotic, and ER stress responses via pathways such as MAPK and Akt. The extent of oxidative stress is commonly evaluated using biomarkers including ROS, malondialdehyde (MDA), superoxide dismutase (SOD), and glutathione (GSH) (Fan et al., 2019; Li et al., 2025; Qi et al., 2026; Yi et al., 2019). The antioxidant system is a sophisticated evolutionarily shaped defense network, consisting of endogenous enzymatic components (e.g., SOD, CAT, GPx), non-enzymatic factors (e.g., GSH), and exogenous dietary antioxidants (e.g., vitamin C/E, tea polyphenols, curcumin). Its primary role is to eliminate excess ROS, sustain redox balance, and maintain tissue integrity, with its dynamic equilibrium being essential for protection against oxidative injury, aging retardation, and disease modulation (Liu et al., 2026; Sun et al., 2026).

#### 4.2.1 Regulation of vesicle trafficking

Vesicle trafficking serves as a transmitter of oxidative stress signals. The small GTPase Rab5 is a core component of vesicle trafficking and a key regulator of membrane transport. GDP dissociation inhibitor (GDI), one of the three critical regulatory proteins controlling Rab5 activity, is modulated by p38 MAPK to regulate endocytic membrane transport (Yuan and Song, 2020). Felberbaum-Corti et al. (2007) found that TXNL1 can bind to p38 MAPK, suggesting that TXNL1 may selectively regulate Rab5-mediated fluid-phase endocytosis through the p38 MAPK-GDI pathway under oxidative stress conditions (Felberbaum-Corti et al., 2007). Based on these findings, the researchers hypothesized that TXNL1 may act as a redox sensor, coupling intracellular redox signals to specific membrane trafficking events. However, this hypothesis requires further evidence for validation.

#### 4.2.2 Response to metal oxidants

Metal/metalloid oxidants such as arsenite can induce intracellular oxidative stress. TXNL1 is closely involved in arsenite-induced carcinogenesis (Arkinson et al., 2025; Gao et al., 2025; Wiseman et al., 2009; Zhao et al., 2025). As discussed above, under arsenite-induced oxidative stress conditions, TXNL1 can be degraded by the proteasome via a ubiquitin-independent pathway (Arkinson et al., 2025; Gao et al., 2025). Another independent study (Zhao et al., 2025) revealed an arsenic-induced ubiquitin-dependent mechanism of TXNL1, in which arsenic exposure upregulates the expression of DNA methyltransferase 1 (DNMT1), which in turn induces hypermethylation of the promoter region of the deubiquitinase *USP10* gene (ubiquitin-specific peptidase 10), thereby suppressing *USP10* transcription and protein expression. The reduction in USP10 levels weakens its deubiquitination-mediated protection of TXNL1 (thioredoxin-like protein 1), leading to the accumulation of ubiquitinated TXNL1 and its subsequent degradation via the classical ubiquitin-proteasome pathway. As a critical redox-active protein, TXNL1 depletion significantly impairs the cellular capacity to scavenge reactive oxygen species (ROS), resulting in excessive ROS accumulation, DNA oxidative damage, and malignant transformation of bronchial epithelial cells (such as BEAS-2B). Conversely, exogenous enhancement of TXNL1 expression effectively inhibits the aforementioned arsenic-induced oxidative damage and transformation. This DNMT1–USP10–TXNL1 regulatory axis has been proposed as a core molecular mechanism by which arsenic disrupts cellular redox homeostasis, and also suggests that maintaining TXNL1 stability or intervening in this axis (e.g., by targeting DNMT1 or restoring USP10 function) may serve as a potential strategy for preventing arsenic-induced bronchial carcinogenesis.

This indicates that under conditions of arsenic exposure, TXNL1 degradation may proceed via two distinct mechanistic routes: ubiquitin-dependent and ubiquitin-independent degradation. Nonetheless, several critical questions remain unanswered: firstly, whether cross-talk exists between these two pathways (for instance, whether the status of USP10 influences TXNL1–19S association) and whether a single “molecular switch” controls pathway selection; secondly, how the nature and intensity of stress—specifically, whether acute or chronic metal/metalloid oxidative stress preferentially engages one degradative route over the other; and thirdly, whether mutations at the redox-active sites of TXNL1 modulate its degradation kinetics.

#### 4.2.3 Response to H_2_O_2_

H_2_O_2_, as a classical oxidative stress inducer, directly elevates intracellular ROS levels and activates downstream signaling (Fan et al., 2019). In *Schizosaccharomyces pombe*, Txl1 does not resist oxidative stress by directly scavenging H_2_O_2_. Instead, Txl1 utilizes reversible changes in its redox state to act as a negative regulatory element, modulating the transmission of H_2_O_2_ signals to transcriptional responses (Brown et al., 2013).

## 5 TXNL1 and disease

TXNL1 has been implicated in a broad spectrum of clinical conditions, including cancer, ischemic brain injury, and spinal cord injury (Table 1). Among these, the most comprehensive mechanistic insights come from studies on arsenic-induced lung carcinogenesis. Notably, alterations in TXNL1 expression or function have also been documented in other disease contexts, though the precise mechanisms remain largely undefined.

**TABLE 1:** Overview of TXNL1 in clinical disease research.

Specific disease	TXNL1 expression/Functional change	Mechanism and clinical significance	Key references
Gastric cancer (cisplatin resistance)	Significantly downregulated in cisplatin-resistant cell lines (BGC823/DDP, SGC7901/DDP)	*TXNL1* negatively regulates *XRCC1* expression via the ubiquitin-proteasome pathway; overexpression of TXNL1 induces apoptosis through Bcl-2-mediated mitochondrial pathway, reversing cisplatin resistance	Xu et al. (2014)
Colorectal cancer	Differentially expressed in aneuploid colorectal cancer; serves as a potential marker to distinguish aneuploid from diploid tumors along with HDAC2	Overexpression of TXNL1 may enhance its transcriptional repression activity through binding to the transcription factor B-Myb, thereby specifically inducing cell cycle arrest at the G0/G1 phase and maintaining genomic stability	Gemoll et al. (2011)
Lung cancer (arsenic-induced)	Downregulated specifically upon arsenic exposure (both *in vivo* and *in vitro*)	Arsenic disrupts redox homeostasis via the *DNMT1-USP10-TXNL1* axis: arsenic upregulates *DNMT1* → promoter hypermethylation of *USP10* → transcriptional repression of *USP10* → increased ubiquitin-dependent degradation of TXNL1 → elevated ROS production and DNA oxidative damage → promotes malignant transformation	Zhao et al. (2025)
Ischemic brain injury	Tat-TXNL1 fusion protein attenuates brain ischemic injury (exogenous supplementation)	Tat-TXNL1 penetrates cell membranes and exerts neuroprotective effects by: a. inhibition of reactive oxygen species (ROS) production and attenuation of neuroinflammatory responses; b. modulation of MAPK and Akt signaling cascades, regulation of pro-apoptotic protein expression, and mitigation of hippocampal neuronal death	Cha et al. (2023)
Spinal injury	Upregulated (as a downstream effector of the miR-30a-5p/*Neurod1* axis)	miR-30a-5p inhibits Neurod1, upregulating TXNL1, SEPN1, and GPX1, which suppresses inflammation and enhances oxidative stress resistance. TXNL1 serves as a downstream effector of this protective axis	Fu et al. (2018)

### 5.1 Gastric cancer

Cisplatin is a cornerstone chemotherapeutic agent for gastric cancer, yet acquired resistance remains a major obstacle leading to treatment failure. XRCC1 (X-ray repair cross-complementing protein 1) is a key scaffolding protein for DNA single-strand break (SSB) repair and serves as a central hub in the DNA damage repair network, playing a critical role in maintaining genomic stability. In cancer therapy, high expression of XRCC1 often indicates enhanced DNA repair capacity in tumor cells, thereby conferring resistance to DNA-damaging chemotherapeutic agents such as platinum-based drugs. A study has shown that TXNL1 negatively regulates XRCC1 expression in the gastric cancer cell line BGC823 and in gastric cancer tissues, ultimately enhancing the sensitivity of gastric cancer cells to cisplatin (Xu et al., 2014).

### 5.2 Colorectal cancer

TXNL1 is significantly overexpressed in euploid colorectal cancer cell lines and clinical samples while markedly reduced in aneuploid colorectal cancer (Gemoll et al., 2011). It has been postulated that overexpression of TXNL1 may enhance its transcriptional repression activity through binding to the transcription factor B-Myb, thereby specifically inducing cell cycle arrest at the G0/G1 phase and maintaining genomic stability (Kim et al., 2006). High expression of TXNL1 helps euploid cancer cells preserve genomic stability, whereas low expression correlates with high invasiveness and poor prognosis in aneuploid tumors (Gemoll et al., 2011). Therefore, TXNL1 may serve as a potential biomarker for distinguishing molecular subtypes of colorectal cancer.

### 5.3 Carcinogenic transformation of bronchial epithelial cells

Arsenic, as an oxidative stress trigger, promotes malignant transformation of bronchial epithelial cells. In contrast to most antioxidant genes that are upregulated upon stimulation, arsenic selectively downregulates TXNL1 expression. The loss of TXNL1 impairs the cellular capacity to scavenge reactive oxygen species (ROS), leading to excessive ROS accumulation and DNA oxidative damage, which ultimately drives malignant transformation of bronchial epithelial cells (Zhao et al., 2025). Thus, TXNL1 serves as a critical antioxidant that effectively interrupts the arsenic-induced damage cascade, conferring essential protection against malignant transformation of bronchial epithelial cells. Maintaining TXNL1 expression holds significant potential for the prevention of arsenic-induced lung cancer.

### 5.4 Ischemic brain injury

Ischemia-reperfusion, as a pathological process, aggravates tissue injury via an overwhelming burst of reactive oxygen species (Qi et al., 2026). During ischemic brain injury, excessive activation of MAPK and apoptotic signaling pathways induced by oxidative stress is a key process leading to neuronal death. Targeting this pathological mechanism, a study has found that a cell-permeable fusion protein, Tat-TXNL1, can effectively penetrate cell membranes (Cha et al., 2023). The exogenously delivered cell-permeable fusion protein Tat-TXNL1 exhibits clear neuroprotective effects. In hydrogen peroxide-treated hippocampal neuronal cells (HT-22), Tat-TXNL1 effectively suppresses reactive oxygen species (ROS) production and cell death, modulates the activation of MAPK and Akt signaling pathways, and regulates the expression levels of pro-apoptotic proteins. In ischemic animal models, this protein significantly reduces hippocampal neuronal death, while inhibiting the activation of astrocytes and microglia, thereby alleviating neuroinflammatory responses. The study concludes that Tat-TXNL1, through its combined intervention in oxidative stress, apoptotic signaling, and neuroinflammation, holds potential as a therapeutic protein for ischemic brain injury.

Notably, the role of TXNL1 in apoptosis regulation may vary depending on cell type and pathological context. In Newcastle disease virus (NDV)-infected DF-1 fibroblasts, the V protein of NDV directly interacts with TXNL1. Overexpression of TXNL1 induces apoptosis in DF-1 cells and suppresses NDV replication, whereas knockdown of TXNL1 attenuates apoptosis. The V protein inhibits apoptosis by interfering with TXNL1 function, thereby creating conditions favorable for viral replication (Wang et al., 2018).

### 5.5 Spinal injury

Excessive oxidative stress and inflammatory responses following spinal cord injury constitute a major pathological barrier to neurological recovery. A study has found that miR-30a-5p, by targeting and inhibiting neurogenesis differentiation factor 1 (Neurod1), ultimately upregulates the expression of multiple antioxidant proteins—including TXNL1, SEPN1, and GPX1-within the injured area (Fu et al., 2018). This molecular cascade effectively suppresses the inflammatory response and enhances neuronal resistance to oxidative stress. In this study, TXNL1 serves as a functional effector molecule downstream of the miR-30a-5p/Neurod1 signaling axis, participating in the execution of neuroprotection. Its upregulation is regarded as an important indicator of the successful activation of this protective pathway.

In summary, TXNL1 demonstrates promising potential for targeted therapeutic intervention. The functional responses of TXNL1 are highly context-dependent, varying across different disease or stress conditions. In brain and spinal cord injury, TXNL1 is mobilized to exert neuroprotective functions. In gastric and colorectal cancers, reduced expression or functional loss of TXNL1 correlates with drug resistance and enhanced tumor aggressiveness. With respect to apoptosis regulation, TXNL1 displays opposing roles: Tat-TXNL1 confers anti-apoptotic effects in ischemic brain injury models, whereas in NDV-infected DF-1 fibroblasts, TXNL1 promotes apoptosis. This marked difference suggests that the function of TXNL1 may be context-dependent, potentially regulated by the specific type of stress and the cellular microenvironment. However, it must be emphasized that this classification remains descriptive in nature and does not yet reveal the molecular switches or upstream signaling mechanisms that determine its functional polarity—a critical gap that warrants urgent investigation. Based on the structural features of TXNL1, we speculate that its functional transition may be governed by stress-induced changes in cellular redox status, or alternatively, may result from the activation of distinct ubiquitin-independent degradation pathways triggered by different levels of oxidative damage. Nevertheless, these hypotheses require rigorous experimental validation through site-directed mutagenesis and interactomics-based approaches. Elucidating these regulatory mechanisms is a key step toward elevating TXNL1 from a mere phenotype-associated factor to a mechanistic node with therapeutic intervention value.

## 6 Summary

In summary, TXNL1 participates in two fundamental biological processes—redox regulation and protein quality control—and holds significant clinical relevance as a redox hub, a disease biomarker, and a therapeutic target. As a member of the antioxidant system, TXNL1 plays a protective role in arsenic-induced lung cancer and its downregulation in cisplatin-resistant gastric cancer suggests its potential as a predictive biomarker. Its neuroprotective effects in ischemic and spinal cord injury further underscore its therapeutic potential. However, numerous key questions remain to be addressed.

Future research should focus on: (a) elucidating its nucleocytoplasmic dynamics and tissue/cell-type specificity using high-resolution localization techniques; (b) clarifying the interplay between its redox and protein quality control functions, with particular emphasis on whether TXNL1, as a conformation-sensitive proteasome cofactor participating in the proteasome regulatory network, promotes the degradation of protein substrates other than itself; and (c) more precisely delineating whether TXNL1 exhibits distinct response mechanisms depending on the type of stress (e.g., oxidative damage vs. viral infection) across different pathological contexts, thereby providing a theoretical basis for targeted intervention.
